# Xanthine oxidase inhibitory kinetics and mechanism of ellagic acid: In vitro, in silico and in vivo studies

**DOI:** 10.1049/nbt2.12135

**Published:** 2023-05-08

**Authors:** Jianmin Chen, Zemin He, Sijin Yu, Xiaozhen Cai, Danhong Zhu, Yanhua Lin

**Affiliations:** ^1^ School of Pharmacy Putian University Putian Fujian China; ^2^ Key Laboratory of Pharmaceutical Analysis and Laboratory Medicine (Putian University) Fujian Province University Putian Fujian China

**Keywords:** biochemistry, biological techniques, drugs, enzymes

## Abstract

Ellagic acid (EA), which is widely distributed in many foods, has been found to possess inhibitory activity against xanthine oxidase (XO). However, there is ongoing debate about the difference in XO inhibitory activity between EA and allopurinol. Additionally, the inhibitory kinetics and mechanism of EA on XO are still unclear. Herein, the authors systematically studied the inhibitory effects of EA on XO. The authors’ findings showed that EA is a reversible inhibitor with mixed‐type inhibition, and its inhibitory activity is weaker than allopurinol. Fluorescence quenching experiments suggested that the generation of EA‐XO complex was exothermic and spontaneous. In silico analysis further confirmed that EA entered the XO catalytic centre. Furthermore, the authors verified the anti‐hyperuricemia effect of EA in vivo. This study elucidates the inhibition kinetics and mechanism of EA on XO, and lays a theoretical foundation for the further development of drugs and functional foods containing EA for the treatment of hyperuricemia.

## Graphical abstract

Inhibitory kinetics and mechanism of ellagic acid on xanthine oxidase were evaluated by using UV spectra, fluorescence spectra, molecular docking and the hyperuricemia mice model.

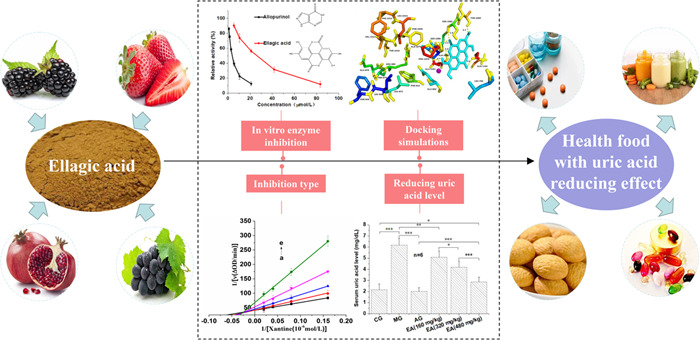

## 1 INTRODUCTION

Ellagic acid (EA) is a natural polyphenol that was first discovered in 1831 [1]. It is found in free from or in the form of ellagitannins in various foods, including strawberries, raspberries, plums, pomegranates, longan seeds, almonds, and walnuts [2]. With four hydroxyl groups, two lactones and two hydrocarbon rings, EA possesses exceptional antioxidant properties and exhibits a wide range of pharmacological activities. Researches have demonstrated that EA has hepatoprotective [3], cardioprotective [4], anti‐anxiety [5], anti‐diabetic [6], anti‐bacterial [7], anti‐viral [8], and anti‐cancer [9] properties. Considering these numerous health benefits, it is apparent that EA is a crucial compound for maintaining good health and preventing disease [10]. Consequently, EA has been gaining increasing attention in scientific research and biomedical applications. Recent advances in biological functionalities, production and applications of EA have been well‐summarised [11, 12].

Moreover, EA isolated from plant extracts has recently been identified as an effective inhibitor of xanthine oxidase (XO) [13, 14]. However, the literature on the actual XO inhibitory activity of EA is confusing, with conflicting IC_50_ values reported. One study reported that EA had an IC_50_ (concentration required to inhibit 50% enzyme activity) value of 3.1 μmol/L, indicating weaker inhibitory activity than allopurinol (0.17 μmol/L) [15]. Similarly, another study reported that the IC_50_ values of EA and allopurinol were 71.5 and 10.4 μmol/L, respectively [16]. However, a later study reported an IC_50_ of 18 μmol/L for EA, which was better than allopurinol (52 μmol/L) [17]. In another study, the IC_50_ values of EA and allopurinol were determined to be 6.5 and 28.6 μmol/L, respectively [18]. The results of the last two studies were inconsistent with previous studies. A more recent study showed that the inhibitory effect of EA (165.6 μmol/L) was much weaker than allopurinol (6.733 μmol/L) [19]. These results create confusion about the actual XO inhibition activity of EA and its potential to replace allopurinol, which has serious side effects [20].

Therefore, we hypothesise that EA's inhibitory effects on XO require further investigation. In this study, we comprehensively reassess the inhibitory activity of EA on XO in vitro, including studying its inhibition kinetics, and mechanism through ultraviolet spectroscopy, fluorescence quenching, and molecular docking technology. We also evaluate the effect of EA on reducing uric acid in vivo using an animal model of hyperuricemia. These findings are crucial for the development of functional foods and drugs for the treatment of hyperuricemia and gout.

## 2 MATERIALS AND METHODS

### 2.1 Materials

Xanthine oxidase (XO, cow milk, 35.7 U/mL) and xanthine (>99.5%) were purchased from Sigma‐Aldrich (Shanghai, China). Ellagic acid (EA, >98.0%), allopurinol, uric acid, potassium oxonate (PO), lithium sulphate, phosphotungstic acid and dimethyl sulfoxide (DMSO) were obtained from Aladdin Bio‐Chem Technology Co., LTD (Shanghai, China). And the other reagents and solvents used in the experiments were of analytical or chromatographic grade unless otherwise stated.

### 2.2 Determination of xanthine oxidase activity

Xanthine oxidase activity was determined following a previously reported method [20]. The test compounds were dissolved in DMSO, and the resulting solutions were appropriately diluted for the assay. Xanthine oxidase and xanthine solutions with concentrations of 0.04 U/mL and 0.1 mmol/L, respectively, were prepared using 0.2 mol/L phosphate buffered solution (PBS, pH 7.4) as the solvent. An aliquot of the test solution (0.1 ml) with various concentrations was added separately to XO solution (0.2 ml). The resulting solution was well mixed and incubated for 5 min at 298 K. Then, the substrate (xanthine) solution was pipetted into the mixture to initiate the enzymatic reaction, and the OD value of the resulting solution was recorded every 60 s at 290 nm using a UV spectrometer (UV 2550, SHIMADZU) at 298 K. Xanthine oxidase activity was determined without adding an inhibitor and was considered 100%. Allopurinol and DMSO were used as positive and negative controls, respectively, for the commonly used XO inhibitor and solvent. Each test was performed three times (*n* = 3). The equation for calculating the relative activity (RA) of XO is as follows: RA (%) = *R*_i_/*R*_c_ × 100%, where *R*_i_ and *R*_c_ represent the reaction rates of the solution with and without inhibitors, respectively. The IC_50_ value was calculated based on the plot of RA versus inhibitor concentration.

### 2.3 Kinetic analysis

To perform a kinetic analysis of the inhibitory activity of EA on XO, the enzymatic reaction rates were determined in the presence of various concentrations of EA (0, 5.21, 10.42, 20.83 and 41.67 μmol/L) with xanthine concentrations ranging from 6.25 to 50 μmol/L. The Lineweaver–Burk equation (Equation (1), double reciprocal form) was used to determine the type of inhibition as follows [21]:

(1) $$\frac{1}{\nu }=\frac{K_{m}}{V_{\max}}\left(1+\frac{\left[I\right]}{K_{i}}\right)\frac{1}{\left[S\right]}+\frac{1}{V_{\max}}\left(1+\frac{\left[I\right]}{K_{is}}\right)$$

For further analysis of the data, the following equations were used:

(2) $$Slope=\frac{K_{m}}{V_{\max}}+\frac{K_{m}\left[I\right]}{V_{\max}K_{i}}$$

(3) $$Y-intercept=\frac{1}{V_{\max}}+\frac{1}{{K_{is}V}_{\max}}\left[I\right]$$

Here, [S] and [I] represent the substrate (xanthine) and inhibitor (EA) concentrations, respectively. The reaction rate and maximum reaction rate are represented by ν and V_max_, respectively. *K*_*i*_ and *K*_*is*_ indicate the inhibition constants of EA binding with the free XO and XO‐xanthine complex, respectively. The Michaelis‐Menten constant is denoted by the symbol *K*_*m*_. The type of inhibition exerted by EA on XO was determined using Equation (1). The inhibition constants *K*_*i*_ and *K*_*is*_ were calculated using the second curves (based on Equations (2) and (3)) of the apparent *K*_*m*_/V_max_ and 1/V_max_ versus [I], respectively.

### 2.4 Fluorescence titration assay

An aliquot of XO solution (2.0 ml, 0.04 U/mL) was placed in a quartz cuvette (1 cm) and titrated with 0.1 ml EA solution (0.1 mmol/L) each time. The mixture was then placed in a thermostatic bath, and after 5 min, fluorescence spectra ranging from 290 to 500 nm (excitation: 280 nm) were recorded at 298, 304 and 310 K using the FS5 spectrofluorometer (Edinburgh Instruments, England). The fluorescence background was corrected by subtracting the PBS blank. Additionally, to account for EA's UV absorption, all fluorescence data were corrected according to the following equation:

(4) $$F_{c}=F_{m}e^{\left(A_{1}+A_{2}\right)/2}$$

Here, *F*_*m*_ and *F*_*c*_ represent the measured and corrected fluorescence, respectively, and A_1_ and A_2_ denote EA UV absorbance at 280 nm (excitation wavelength) and 404 nm (emission wavelength), respectively.

### 2.5 Simulation studies of the binding mode

The binding mode between EA and XO was investigated using AutoDock (version 4.2.6). The XO structure file (PDB ID: 1FIQ) was downloaded from the protein database and prepared using AutoDock tools (ADT, version 1.5.4). EA's 3D structure file was obtained from PubChem, and the ligand's rotatable bonds were assigned using ADT. A grid box (90 Å × 90 Å × 120 Å) with a grid spacing of 0.375 Å was set up to encompass the entire XO catalytic centre. Lamarckian genetic algorithm was used to perform the calculations, with running times of 100. The docking conformation with the lowest binding energy among all clusters and/or the largest cluster was considered the optimal conformation, which was further processed and analysed using the PyMOL (version 2.3.0) molecular graphics system. Furthermore, hydrophobic interactions between EA and XO were described using LigPlot^+^ (version 1.4.5) software.

### 2.6 Establishment of hyperuricemic mouse model

ICR mice weighing 25 ± 2 g were purchased from Wushi Dongwu Co., LTD (Fujian, China). The protocol for animal experiments (#202030) was approved by the Animal Use and Care Committee of Putian University. To establish a hyperuricemic mouse model, we followed our previously reported method [20], which involved intraperitoneal injection of PO at a dose of 250 mg/kg. Thirty‐six mice were acclimated for at least 1 week before the experiment and were then randomly divided into 6 groups (*n* = 6): control group (CG), model group (MG), allopurinol group (AG, 20 mg/kg), and EA groups (EAGs, doses 160, 320 or 480 mg/kg for each group, suspended at 0.5% sodium carboxymethylcellulose). Mice in all groups except the CG were given PO to raise serum uric acid levels, while the CG mice were given 1 ml of 0.9% NaCl. After 1 hour, AG and EAGs mice were given allopurinol and EA intragastrically, respectively, while the CG mice were given saline intragastrically. After 2 hours, blood was obtained from the tail vein and was centrifuged at 4000 rpm for 8 min. The serum obtained was kept at −80 °C for the following assay. Uric acid levels in serum were detected using a previously reported method [20].

### 2.7 Statistical analysis

Data were processed using SPSS software (version 20.0, United States) and presented as mean ± standard derivation (SD). A paired *t*‐test analysis was performed to compare the differences between two groups, while One‐way Analysis of Variance was used to compare multiple treatment groups. *P* < 0.05, <0.01 or <0.001 were considered significant differences.

## 3 RESULTS AND DISCUSSION

### 3.1 Inhibition of xanthine oxidase activity by Ellagic acid

**FIGURE 1:**
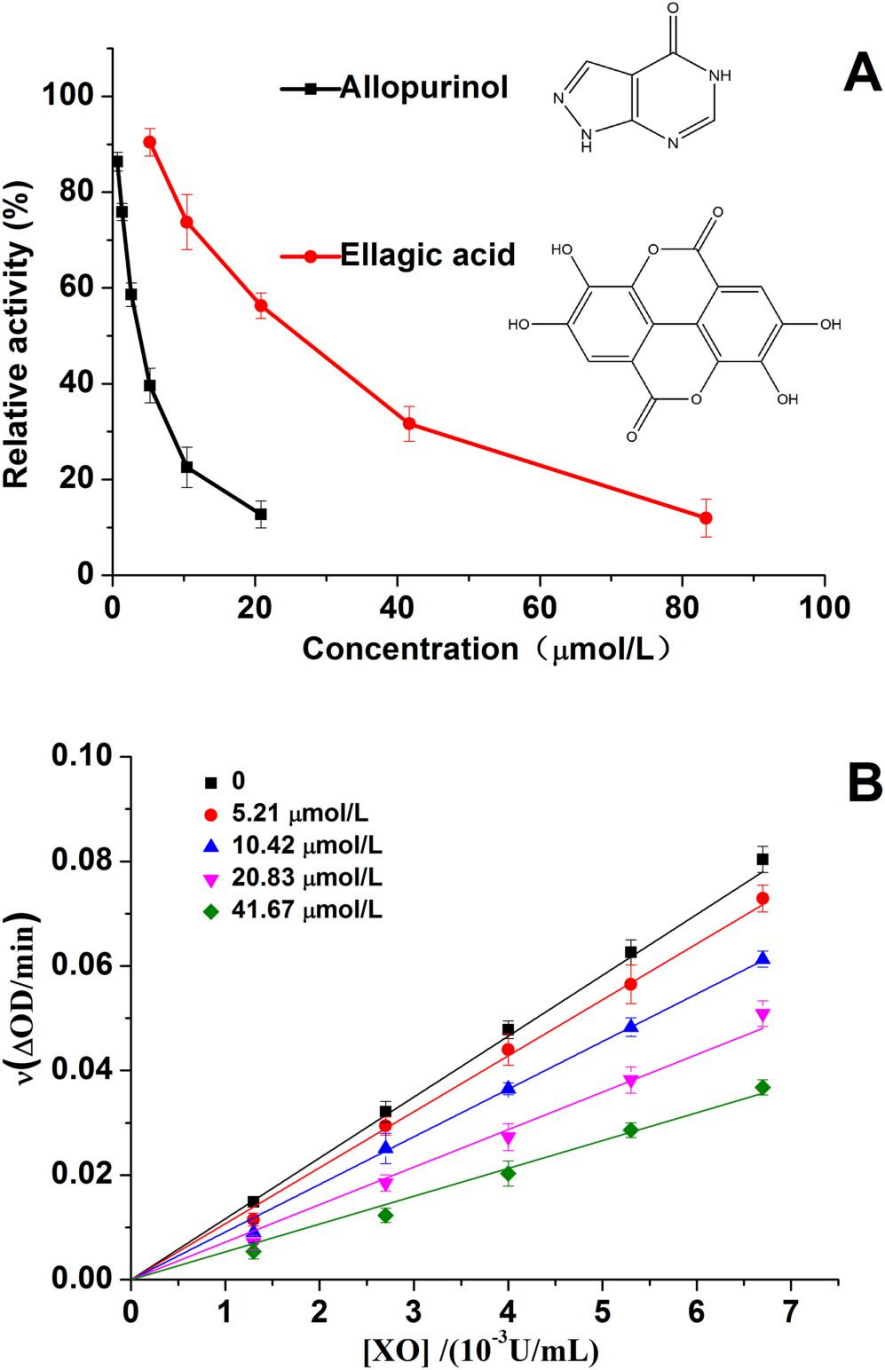
(a) Ellagic acid (EA) and allopurinol inhibitory activities against xanthine oxidase (XO) (*T* = 298 K, pH 7.4); [xanthine] = 0.1 mmol/L, [XO] = 0.04 U/mL. (b) Curves of ν versus [XO]; [xanthine] = 0.1 mmol/L, [EA] = 0, 5.21, 10.42, 20.83 and 41.67 μmol/L for curves a → e, respectively. Data are presented as mean ± SD (*n* = 3).

The effects of EA and allopurinol on XO were investigated through in vitro experiments, and the results are presented in Figure 1a. The XO activity showed a decreasing trend with increasing inhibitor concentration, indicating that both EA and allopurinol could significantly inhibit XO activity in a dose‐dependent manner. Based on the curves, the IC_50_ values of EA and allopurinol were calculated to be 22.97 ± 0.12 and 3.57 ± 0.06 μmol/L, respectively. This suggests that inhibitory effect of EA on XO activity is weaker than that of allopurinol, which is consistent with some previously reported studies [15, 16, 19]. However, other studies have come to the opposite conclusion, which has created confusion regarding the inhibitory capacity of EA compared to allopurinol. Therefore, one of the aims of this study is to clarify this issue.

Upon comparing the methods and steps used in these studies, it was found that the main factor leading to inconsistent experimental results was the difference in the technical methods used. For example, while traditional XO inhibition assays were based on UV spectroscopy [15, 16, 19], one study used fluorescence quenching technology instead [18]. Furthermore, variations in test conditions such as reagent and enzyme concentrations, pH of the buffer solution, reaction temperature, and time could also lead to inconsistent experimental results. In other words, the IC_50_ value can be easily influenced by detection methods, test conditions, and reaction time [22]. These findings highlight the importance of carefully considering technical methods and experimental conditions when conducting research in this area. Therefore, this study employed the traditional UV‐based assay and well‐controlled experimental conditions. Based on the literature and our experimental results, it was concluded that EA's inhibition of XO was somewhat weaker than that of allopurinol, which was also confirmed by the following animal studies. However, EA has been reported to be safe for rats, even with dietary supplements of up to 5% (3011 mg/kg/day) [23]. The results suggest that EA is a promising compound for the treatment of hyperuricemia and gout.

The effect of different amounts of EA on XO activity at various concentrations was determined at a fixed concentration of substrate xanthine (Figure 1b). The plots of ν versus [XO] showed good linearity, and the lines intersected at the origin. Additionally, the slope of the lines was inversely proportional to the amounts of EA, suggesting that EA was a reversible inhibitor of XO [22]. These results indicated that EA may interact with XO in a non‐covalent manner by forming a reversible inhibitor‐enzyme complex, but it would not induce complete inactivation of XO.

### 3.2 Type of Ellagic acid inhibition on xanthine oxidase

**FIGURE 2:**
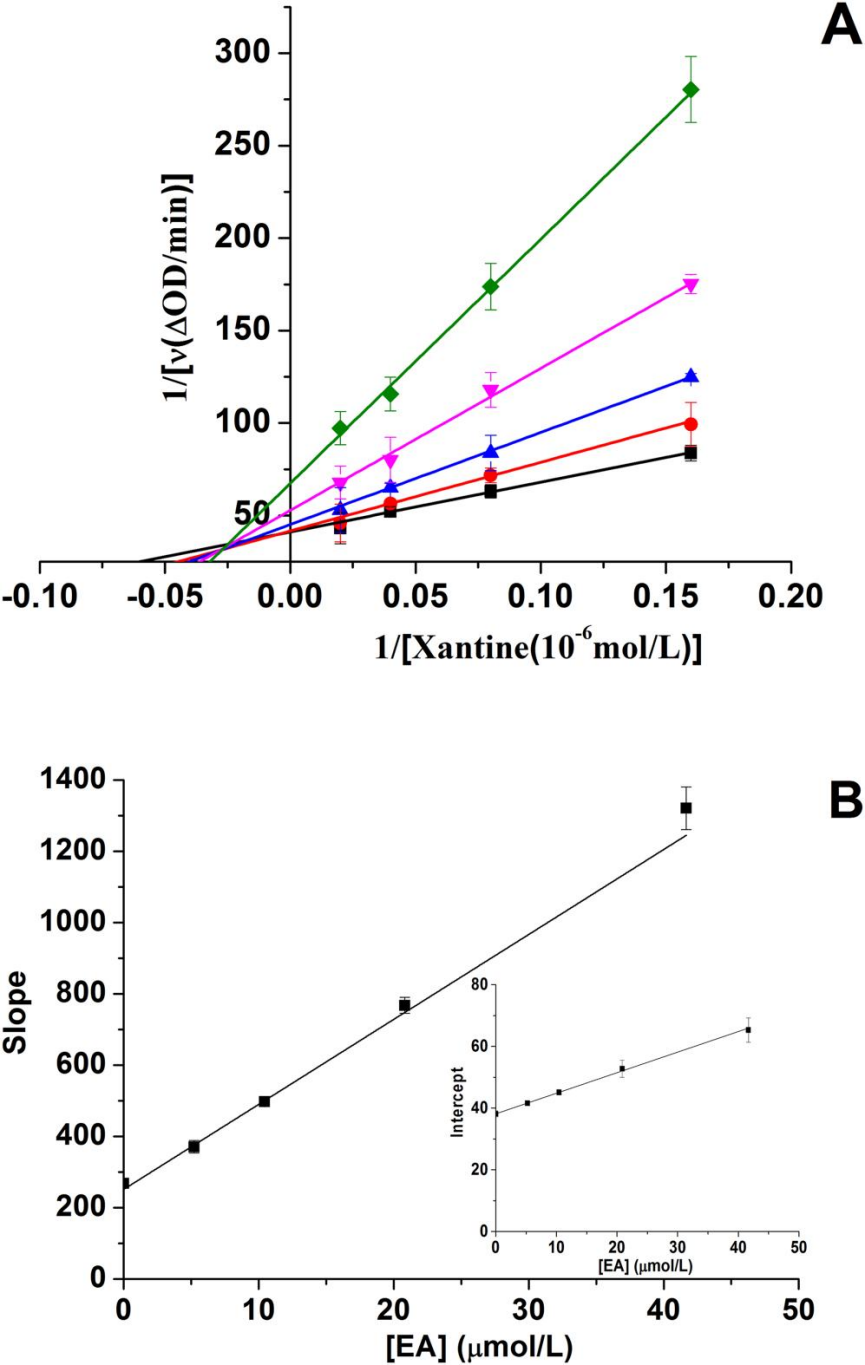
(a) Lineweaver‐Burk plots; [xanthine oxidase (XO)] = 0.04 U/mL, [Ellagic acid (EA)] = 0, 5.21, 10.42, 20.83 and 41.67 μmol/L for curves a → e, respectively. (b) Secondary plots of the slope and the Y‐intercept (inset) versus [EA]. Data are presented as mean ± SD (*n* = 3).

The type of EA inhibition on XO was confirmed by the Lineweaver‐Burk plot. The results (Figure 2a) showed that the fitted lines intersected in the second quadrant. Moreover, both horizontal and vertical intercept values showed an increasing trend with the increasing concentration of EA. These results suggested that EA was a mixed‐type inhibitor of XO, indicating that it may bind to both free XO and XO‐xanthine complexes. The slope and intercept of the plots (based on Equations (2) and (3)) on [EA] were linearly fitted (Figure 2b), indicating that it had one or one class of inhibition sites on XO [24]. Based on the plots mentioned, *K*_*i*_ and *K*_*is*_ were determined to be 10.6 ± 0.08 and 57.24 ± 0.23 μmol/L, respectively. The *K*_*i*_ value was much smaller than *K*_*is*_, indicating that EA was more likely to bind free XO than XO‐xanthine complex [25]. This study demonstrated the type of EA inhibition on XO for the first time, which may help to understand the inhibitory mechanism of EA on XO.

### 3.3 Fluorescence quenching of Ellagic acid on xanthine oxidase

**FIGURE 3:**
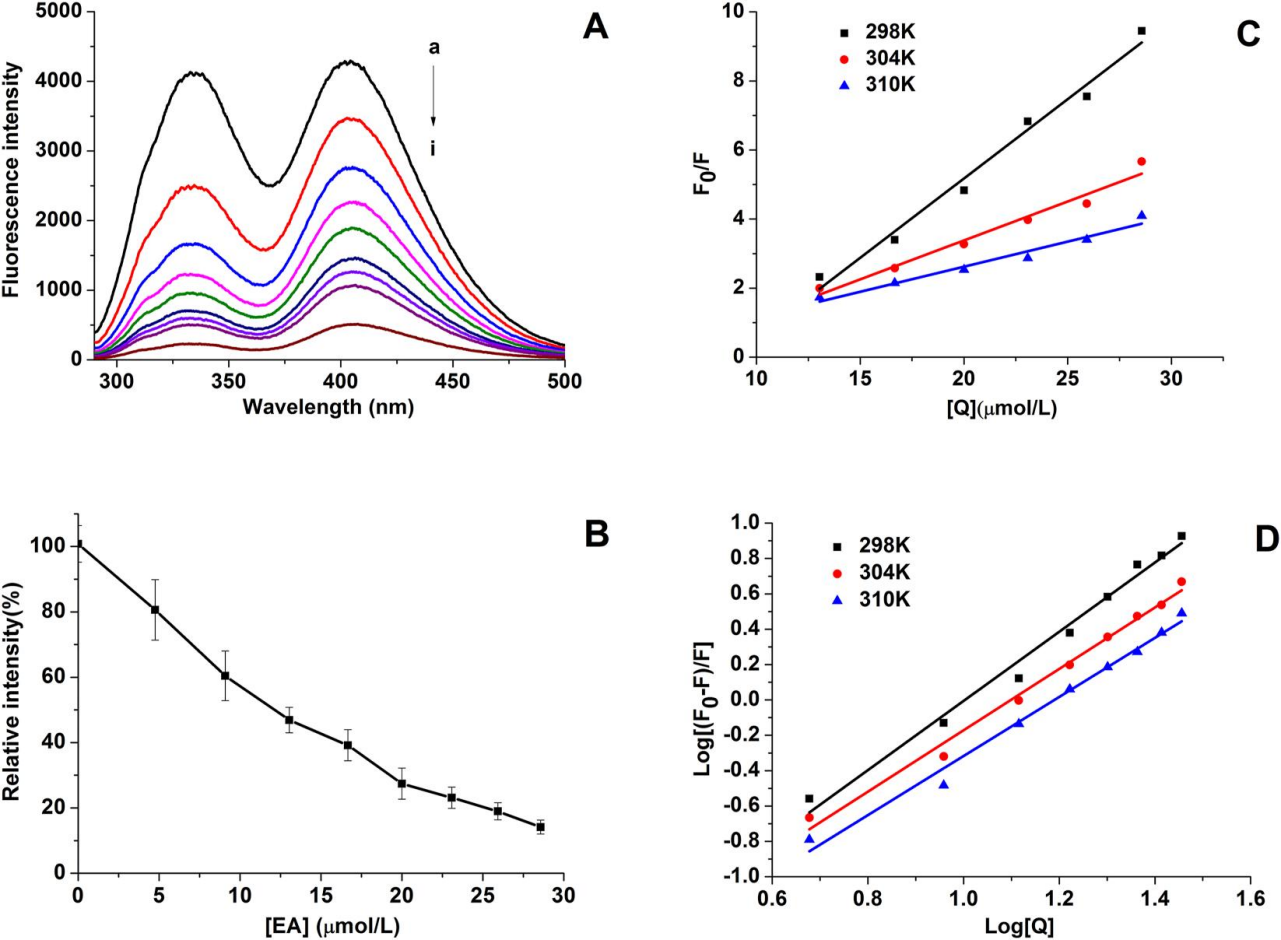
(a) Xanthine oxidase (XO) fluorescence spectra in the presence of Ellagic acid (EA) with different concentrations (pH 7.4, *T* = 298 K, E_x_ = 280 nm); [XO] = 0.04 U/mL, [EA] = 0, 4.76, 9.09, 13.04, 16.67, 20.00, 23.08, 25.92 and 28.57 μmol/L for curves (a)–(i), respectively. (b) Relative fluorescence intensity (%) of XO in the presence of EA. (c) Stern–Volmer plots for studying XO fluorescence quenching by EA at 298, 304 and 310 K. (d) Secondary plots of log [(F_0_ − F)/F] versus log [Q] at 298, 304 and 310 K.

Although kinetic studies have shown considerable inhibitory activity of EA on XO, the binding mode between EA and XO is still unclear. Fluorescence quenching technology has recently been used to evaluate the binding between small molecule compounds and enzymes. Therefore, the fluorescence titration assay was performed to elucidate the binding interaction between EA and XO. The fluorescence emission spectra were recorded and presented in Figure 3a, and two emission peaks (336 and 404 nm) were observed. Fluorescence data at 404 nm were used to further analyse the interaction mode. In Figures 3a and 3b, fluorescence intensity showed a decreasing trend with increasing EA concentration. Specifically, fluorescence intensity decreased to 14.15% when EA concentration reached 28.57 μmol/L. In addition, the maximum emission wavelength remained unchanged. These findings directly demonstrated that the interaction between EA and XO occurred.

The fluorescence quenching mechanism mainly includes dynamic and static quenching. The Stern‐Volmer equation shown below was used to distinguish the quenching mechanism.

(5) $$F_{0}/F=1+K_{q}\tau_{0}\left[Q\right]=1+K_{sv}\left[Q\right]$$

The fluorescence intensities with and without EA are indicated by the symbols *F* and *F*_0_, respectively. The quenching rate constant of the biological macromolecule is represented by *K*_*q*_. The average lifetime of fluorophore is represented by the symbol τ_0_ (10^−8^ s). *K*_SV_ is the dynamic quenching constant of Stern–Volmer; [Q] is the concentration of EA. The curves (Figure 3c) of *F*_0_/*F* versus [Q] at 298, 304 and 310 K showed good linearity, indicating that there was only one quenching process, either static or dynamic quenching [26]. Then *K*_SV_ values at 298, 304 and 310 K were calculated to be 1.881 × 10^5^, 1.661 × 10^5^, and 1.455 × 10^5^ L/mol, respectively, using the Stern‐Volmer equation. These values showed a decreasing trend as the temperature increased. Similarly, the *K*_*q*_ values at 298, 304 and 310 K were determined to be 1.881 × 10^13^, 1.661 × 10^13^, and 1.455 × 10^13^ L/(mol·s), respectively. These values were all significantly larger than the maximum scattering collision quenching constant [2.0 × 10^10^ L/(mol·s)], indicating that a static quenching mechanism was involved in the interaction between EA and XO [26].

### 3.4 Xanthine oxidase binding sites for ellagic acid

For the static quenching process, the following double logarithmic equation was used to calculate *K*_a_ and n, which represent the apparent binding constant and the number of binding sites, respectively.

(6) $$\log\frac{F_{0}-F}{F}=\log\;K_{a}+nlog\left[Q\right]$$

The secondary plots based on the above equation are shown in Figure 3d, from which *K*_a_ and n are obtained (Table 1). All *K*_a_ values were in the order of magnitude of 10^4^ L/mol and decreased with increasing temperature. This suggested that EA had a moderate binding affinity with XO, and the binding interaction was gradually weakened with increasing temperature. At different temperatures, the values of n were all close to 1, indicating that there was only one or one class of XO inhibition sites for EA, which was consistent with the results in Section 3.2 [25].

**TABLE 1:** Kinetic and thermodynamic parameters for the interaction between Ellagic acid (EA) and xanthine oxidase (XO) at 298, 304, and 310 K.

T(K)	K_sv_ (×10^5^ L/mol)	R_a_	K_a_ (×10^4^ L/mol)	R_b_	n	△*H* (kJ/mol)	△*G* (kJ/mol)	△*S* (J/mol/K)
298	1.881 ± 0.05	0.9805	1.67 ± 0.02	0.9936	1.183 ± 0.02	−23.07 ± 0.25	−24.07 ± 0.28	3.36 ± 0.03
304	1.661 ± 0.03	0.9928	1.21 ± 0.03	0.9874	1.236 ± 0.04		−24.09 ± 0.32	
310	1.455 ± 0.04	0.9914	1.17 ± 0.02	0.9921	1.308 ± 0.02		−24.11 ± 0.34	

*Note:* The correlation coefficient for the values of K_SV_ and K_a_ were indicated by the symbol of R^a^ and R^b^, respectively.

### 3.5 Interaction forces between ellagic acid and xanthine oxidase

To determine the interaction forces between EA and XO, such as hydrogen bonding, hydrophobic interaction, van der Waals force, and electrostatic force, thermodynamic parameters including entropy change (△*H*), enthalpy change (△*S*), and Gibbs free energy (△*G*) were analysed using the Van't Hoff Equation (7) and Gibbs‐Helmholtz Equation (8) at different temperatures [27]. The equations are as follows:

(7) $$\log\;K_{a}=-\frac{∆H}{2.303RT}+\frac{∆S}{2.303R}$$

(8) ∆G=∆H−T∆S

Here, K_a_ represents the binding constant at 298, 304 and 310 K, and the universal gas constant [8.314 J/(mol·K)] and temperature (K) are represented by the symbols T and R, respectively. The calculated values of △*H*, △*S* and △*G* are summarised in Table 1. The positive/negative and magnitude values of these thermodynamic parameters can determine the main interaction force of EA‐XO complex. The negative value of △*G* suggested that the binding process of EA and XO was spontaneous. Furthermore, the negative value of △*H* and the positive value of △*S* indicate that hydrogen bonding and hydrophobic interaction were the main driving forces in the formation of EA‐XO complex [20].

### 3.6 In silico analysis of EA‐XO complex

**FIGURE 4:**
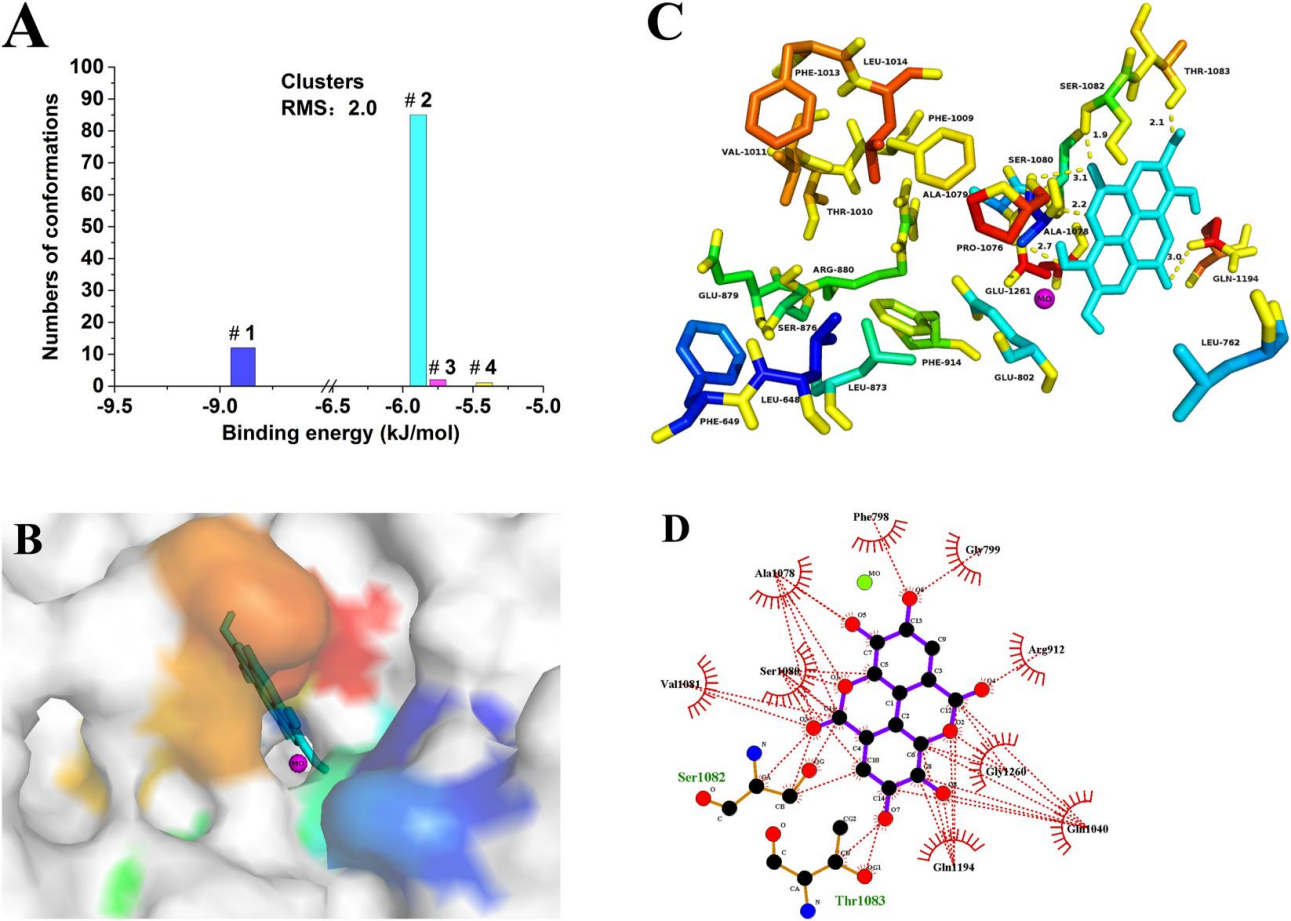
In silico analysis of the EA‐XO complex: (a) Cluster analysis of conformations; (b) Ellagic acid (EA) (the coarser stick in cyan) is inserted into the catalytic site, which is composed of Mo ion (magenta sphere) and numerous amino residues (marked in colour) and presented in surface form; (c) Xanthine oxidase (XO) is presented in ribbon form, and the yellow dashed line stands for hydrogen‐bond; (d) Hydrophobic interactions between EA and XO.

The binding mode between EA and XO was analysed in silico, and the results are shown in Figure 4. ADT was used to cluster all conformations (a total of 100) with a root mean square deviation tolerance of 2.0, resulting in 4 clusters (Figure 4a). The conformation in the largest cluster with the lowest energy was considered optimal. Therefore, the lowest energy (−5.89 kJ/mol) conformation in the second cluster (#2) was identified as the optimal conformation. In Figure 4b, the XO surface structure and optimum conformation are displayed. Ellagic acid (represented by a coarser turquoise stick) was inserted into the catalytic site, which consists of a Mo ion (magenta sphere) and various residues (highlighted in colour), such as GLN‐762, GLU‐802, LEU‐873, SER‐876, GLU‐879, PHE‐914, VAL‐1011, and PHE‐1013 [28]. Once EA was inserted into the catalytic site, the substrate xanthine was blocked outside the catalytic site, inhibiting enzyme activity.

Specifically, EA was inserted into the catalytic site by forming 6 hydrogen bonds (indicated by yellow dashed line) with some residues, including GLN‐1194 (*d* = 3.0 Å), SER‐1082 (*d* = 1.9 Å), THR‐1083 (*d* = 2.1 Å), SER‐1080 (*d* = 2.2 Å), ALA‐1078 (*d* = 3.1 Å), and ALA‐1079 (*d* = 2.7 Å), as shown in Figure 4c. In addition, the results of LigPlot^+^ analysis are shown in Figure 4d. Eleven XO residues were involved in hydrophobic interactions with EA, including THR‐1083, SER‐1082, VAL‐1081, SER‐1080, ALA‐1078, PHE‐798, GLY‐799, ARG‐912, GLY‐1260, GLN‐1040, and GLN‐1194. The results indicated that hydrogen bonds and hydrophobic interaction played a critical role in the binding interaction between EA and XO. These findings were consistent with the results of the fluorescence analysis (Section 3.5). In summary, molecular docking analysis provided insight into the interaction between the ligand and macromolecule, which further clarified EA's inhibitory mechanism on XO.

### 3.7 Anti‐hyperuricemia effect of ellagic acid on mice

**FIGURE 5:**
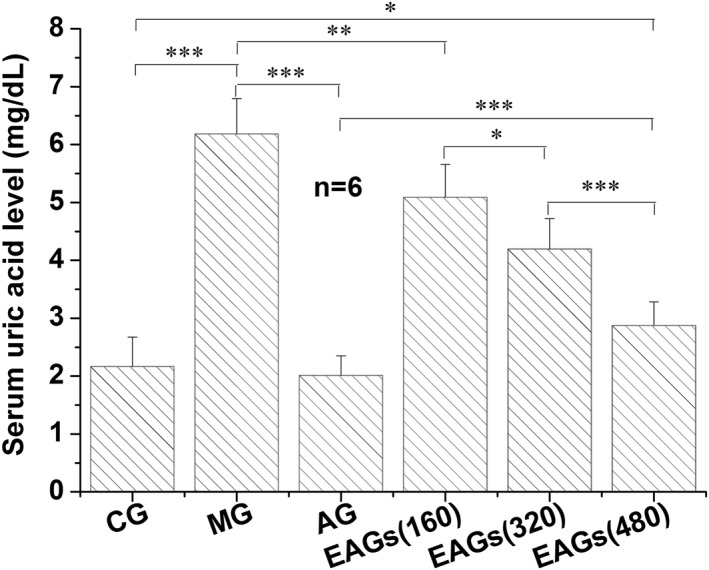
Lower uric acid effects of Ellagic acid (EA) and allopurinol on mice. Control group (CG): mice without any treatment; Model group (MG): mice administered with potassium oxonate (PO); Allopurinol group (AG): mice administered with PO followed by administering 20 mg/kg allopurinol intragastrically; EA groups (EAGs, 160, 320 and 480 mg/kg): mice administered with PO followed by administering different doses of EA intragastrically. Data are presented as mean ± SD. *P < 0.05, **P < 0.01 or ***P < 0.001 were considered significant differences.

The anti‐hyperuricemia effect of EA on mice was evaluated using the PO‐induced hyperuricemia mouse model. As shown in Figure 5, intraperitoneal injection of PO significantly increased uric acid concentration in MG mice (6.18 ± 0.18 mg/dl) when compared to CG mice (2.16 ± 0.51 mg/dl) (*P* < 0.001). Compared with MG, AG serum uric acid (20 mg/kg allopurinol) decreased significantly (*P* < 0.001) to 2.01 ± 0.34 mg/dl, but there was no significant difference with CG (*P* > 0.05). These results demonstrated that the hyperuricemic mouse model was successfully established. After intragastric administration of EA (160, 320 and 480 mg/kg), serum uric acid levels in mice decreased to 5.09 ± 0.57, 4.19 ± 0.53 and 2.87 ± 0.41 mg/dl, respectively, with significant differences compared to MG (*P* < 0.01, *P* < 0.001 and *P* < 0.001), indicating that EA does have the effect of lowering serum uric acid levels. Moreover, there were significant differences among different doses of EAGs (*P* < 0.05), indicating that the uric acid lowering effect of EA was dose‐dependent.

Compared to AG, the anti‐hyperuricemia effect of EA (even at 480 mg/kg) was significantly weaker than that of allopurinol (20 mg/kg). The main reason may be that EA's XO inhibitory activity (IC_50_, 22.97 ± 0.12) was much weaker than allopurinol (IC_50_, 3.57 ± 0.06 μmol/L). The results of in vivo experiments further clarified the confusion of XO inhibitory activity between EA and allopurinol. In addition, another reason may be the very poor bioavailability of EA due to its low solubility, limited permeability, and first pass effect [29]. However, it was reported that the no‐observed‐effect level (NOEL) of EA was 3011 mg/kg/day for rats, and therefore the estimated value for mice was 4215 mg/kg/day [23], which was much higher than the maximum dose (480 mg/kg) administered in this study. These findings demonstrated that EA is an effective and safe candidate compound for the treatment of hyperuricemia and gout.

## 4 CONCLUSION

This study demonstrated that EA is a reversible mixed XO inhibitor with an IC_50_ of 22.97 ± 0.12 μmol/L, which is weaker than that of allopurinol. The mechanism of EA inhibition was explored through in vitro and silico studies, showing that EA formed a static complex with XO through hydrogen‐bond and hydrophobic interactions, hindering substrate entry and ultimately inhibiting enzyme catalytic activity. The animal study demonstrated that EA had an anti‐hyperuricemia effect in mice, although it was weaker than allopurinol, possibly due to its higher IC_50_ value and lower bioavailability. Nevertheless, EA's good drug safety profile suggests its potential as an active compound for treating hyperuricemia and gout. These findings may be useful for developing EA‐rich food nutrition and functional foods for lowering serum uric acid levels.

## AUTHOR CONTRIBUTIONS

Jianmin Chen designed the experiments and wrote the paper. Zemin He, Sijin Yu and Yanhua Lin conducted the experiments. Xiaozhen Cai and Danhong Zhu analysed the data. All authors read and approved the final manuscript.

## CONFLICT OF INTEREST STATEMENT

The authors declare that they have no conflict of interest.

## PERMISSION TO REPRODUCE MATERIALS FROM OTHER SOURCES

None.

## Data Availability

The data that support the findings of this study are available on request from the corresponding author. The data are not publicly available due to privacy or ethical restrictions.
